# Enzyme-Assisted Phenolic Extract of Murtilla Pomace: A Green Food Additive to Prevent Ozone-Induced Oxidation in Salmon

**DOI:** 10.3390/antiox15050593

**Published:** 2026-05-07

**Authors:** Vicente Gregorio Valenzuela-Bass, Elva Gonzales-Nieto, Gabriela Valenzuela-Barra, Miguel Ángel Varas Condori, Angélica Reyes-Jara, Miguel Ángel Rincón-Cervera, Severino Marías de Alencar, Raquel Bridi, Adriano Costa de Camargo

**Affiliations:** 1 Facultad de Ciencias Químicas y Farmacéuticas, Universidad de Chile, Santiago 8380492, Chile; v.valenzuela.4@ug.uchile.cl (V.G.V.-B.); gabriela.m.valenzuela@ciq.uchile.cl (G.V.-B.); 2 Instituto de Nutrición y Tecnología de los Alimentos, Universidad de Chile, Santiago 7830490, Chile; elvagnutricion@gmail.com (E.G.-N.); mvaras@inta.uchile.cl (M.Á.V.C.); areyes@inta.uchile.cl (A.R.-J.); marincer@inta.uchile.cl (M.Á.R.-C.); 3 Department of Agronomy, University of Almería, 04120 Almería, Spain; 4 Department of Food Science and Technology, “Luiz de Queiroz” College of Agriculture, ESALQ/USP, Piracicaba 13418-900, SP, Brazil; smalencar@usp.br; 5 Instituto de Ciencias Aplicadas, Universidad Autónoma de Chile, Santiago 7500910, Chile

**Keywords:** *Ugni molinae* Turcz., waste valorization, polyphenols, lipid oxidation, ozone-induced oxidation

## Abstract

Murtilla pomace (the by-product generated during juice production) shows a high phenolic content. Recovering phenolics from murtilla pomace is a sustainable approach towards zero waste. In this study, murtilla pomace was subjected to enzyme-assisted extraction using Viscozyme. The extracts were analyzed for TPC and phenolic profile. Antioxidant activity was evaluated by chemical-based assays and the antioxidant property was demonstrated for the first time in an ozone-induced oxidation process applied to a raw fish model system. Enzymatic pretreatment with Viscozyme increased the total phenolic content by up to 57%. The antioxidant activity also increased upon enzymatic treatment. The concentration of quercetin was positively affected, while the content of rutin decreased upon enzymatic pretreatment, likely reflecting the enzymatic hydrolysis biotransforming rutin glycoside into quercetin aglycone. Phenolics from murtilla pomace obtained upon enzyme-assisted extraction were shown to be effective as natural antioxidants against ozone-induced oxidation in salmon. Therefore, enzyme-assisted extraction may be an environmentally friendly strategy in the recovery of these natural antioxidants.

## 1. Introduction

The endemic Chilean species *Ugni molinae* Turcz. (Myrtaceae) is of great interest in research because its berries (commonly known as murtilla, murta, or uñi) present the highest content of phenolic compounds among all widely consumed fruits in the southern cone [1]. These compounds confer biological properties that, in some cases, are related to their use in the traditional medicine of the indigenous peoples of southern Chile [2]. Murtilla berries can be consumed raw or used to make products such as infusions, juices, alcoholic beverages, jams, and others [2]. Scientific studies using extracts made from these fruits have demonstrated their antimicrobial [3], antiparasitic [4], analgesic [5], hypoglycemic [6], antioxidant [7], and anti-inflammatory potential [8]. These suggest that murtilla extracts, such as those derived from juices, could present a favorable opportunity for the agroindustry.

Fruits and vegetables exhibit high post-harvest losses ranging from 28% to 55% of total production, which can reach up to 60% in extreme cases [9]. Fruit residues that are not used are also generated during the production of murtilla juice [10]. A significant part of this loss consists of peels, seeds, stems, and other fruit structures separated during the production of juices, jams, and alcoholic beverages [11]. By-products of fruits and vegetables typically end up discarded as waste in landfills, where natural fermentation processes make them a significant source of greenhouse gas emissions [12]. However, they have a wide variety of bioactive compounds, giving them great potential for valorization through their recovery and application [11]. Consequently, various research groups have focused their efforts on studying these residues, with the most extensively studied being orange, lemon, grape, apple, mango, and pineapple juice residues [13,14,15,16]. In this study, our research team has focused on murtilla pomace, the by-product generated during juice production, as a source of phenolic acids and flavonoids [10].

Phenolic compounds have potential applications in nutraceuticals, functional foods, dietary supplements, and/or natural additives [17,18,19,20,21,22,23]. These secondary metabolites can primarily be found in two forms in plant materials: the soluble form (also known as extractable fraction) and insoluble-bound form (non-extractable fraction). The soluble fraction refers to the portion of phenolic compounds that can be extracted from plant material using water or mixtures of water with organic solvents such as methanol, ethanol, or acetone, among others. In contrast, compounds found in the insoluble-bound fraction are chemically bound to macromolecules of the cell wall, such as cellulose, lignin, and structural proteins, and as such cannot be released using these solvents [24]. Compounds in the insoluble-bound form can be recovered using highly alkaline conditions [10] or by applying enzymes that hydrolyze the bonds linking phenolic compounds to macromolecules that impede their release [25].

Using organic solvents in conventional extraction methods has been criticized because most of these solvents are highly flammable, toxic, and harmful to the environment [26]. Furthermore, several organic solvents are not compatible with food applications. Emerging extraction technologies, including enzyme-assisted extraction, have been developed to reduce or eliminate the use of toxic solvents, protecting natural resources and ecosystems [27].

The effect of Viscozyme on the extraction of phenolic compounds from plant materials has been studied in several species, such as grape, apple, soybean, quinoa, and jujube [25,28,29,30], demonstrating its potential for recovering compounds of interest. Additionally, this product can extract phenolic compounds present in the insoluble-bound fraction of plant material when applied to food industry residues, particularly winemaking by-products and wheat bran [25,31]. Based on these studies, Viscozyme is an alternative for obtaining extracts of phenolic compounds that cannot be recovered using the organic solvent mixtures typically employed and as an environmentally friendly option for the valorization of agro-industrial residues.

Several studies demonstrate that ozonated water exhibits strong antimicrobial activity. Ozonation has been found to effectively inhibit growth and inactivate a wide range of microorganisms, including both Gram-positive and Gram-negative bacteria. Therefore, its use in the food industry has been gaining interest and it has gained great importance in seafood products [32]. However, fatty acid oxidation may also occur, especially in products that present a high content of unsaturated fatty acids. This is particularly true for salmon, which is widely recognized as a premium source of omega-3 fatty acids. Furthermore, Chile is the second-largest exporter of Atlantic salmon, surpassed only by Norway [33]. Therefore, the local salmon industry would benefit from identifying natural additives for use in the production of salmon products. In this scenario, natural antioxidants may be used to decrease this process.

Our previous study demonstrated that more than half of the total phenolic compounds present in the residue from murtilla juice, namely murtilla pomace, are in the insoluble-bound form [10]. Therefore, techniques capable of recovering the compounds in this fraction must be sought. This is the first contribution to evaluate the effect of Viscozyme-assisted extraction of murtilla pomace. Likewise, this study evaluates for the first time the effect of Viscozyme-assisted extraction on phenolic recovery from murtilla pomace as a potential food additive to prevent ozone-induced oxidation in salmon.

## 2. Materials and Methods

### 2.1. Materials

Potassium persulfate, DPPH (2,2-diphenyl-1-picrylhydrazyl), ABTS (2,2′-azino-bis(3-ethylbenzothiazoline-6-sulfonic) acid), AAPH (2,2-azobis(2-amidinopropane) dihydrochloride), fluorescein, TPTZ (2,4,6-tris(2-pyridyl)1,3,5-triazine), Trolox^®^ (6-hydroxy-2,5,7,8-tetramethylchroman-2-carboxylic acid), enzymatic mixture Viscozyme^®^, and UPLC-ESI-MS/MS standards were acquired from Sigma-Aldrich Sigma-Aldrich (St. Louis, MO, USA). Sodium hydroxide, fuming hydrochloric acid, glacial acetic acid, ethyl acetate, methanol, HPLC-grade methanol, acetone, iron trichloride, Folin-Ciocalteu reagent, aluminium trichloride, sodium carbonate, sodium acetate, sodium phosphate monobasic, and sodium phosphate dibasic, were acquired from Merck (Darmstadt, Germany).

### 2.2. Methodology

#### 2.2.1. Sample Preparation

Two samples of murtilla fruits were used, both sourced from Chiloé Island, Los Lagos Region, Chile (between 42° S and 43° S latitude and 75° W and 73° W longitude). One of these, referred to as “2022 harvest,” was collected in April 2022, while the other, referred to as “2023 harvest,” was collected in April 2023. The samples were stored at −20 °C immediately after collection. The pedicels, along with any remaining stems and leaves, were manually removed from the fruits. The fruits were then washed under running water. Then, they were blended with distilled water using a Nex BGJ3200 blender (500 W, China) at a ratio of 100 g/L. The resulting juice was filtered through sterile gauze, and the solid residue, composed of seeds, fruit skin fragments, and remnants of the calyx, was recovered. This residue was freeze-dried to complete dryness using FreeZone 2.5 L −50 °C lyophilizer (Labconco, Kansas City, MO, USA), ground into coarse flakes with a mortar, and then ground into a fine powder with a coffee mill CBG5 series coffee mill (Black & Decker, Towson, MD, USA). Thirty grams of this dry powder was blended with 150 mL of hexane for 30 s to remove fats. The hexane was removed through vacuum filtration with e HB eco rotary evaporator (IKA^®^ HB eco, Staufen, Germany), and the process was repeated two additional times. The defatted powder was then weighed using an AS 220/C/2 analytical balance (Radwag, Radom, Poland).

#### 2.2.2. Enzymatic Pretreatment

**Figure 1:**
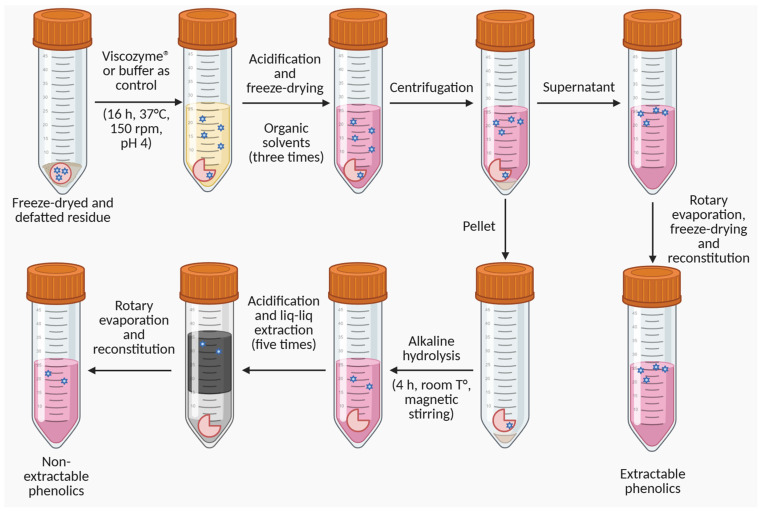
Murtilla Pomace Extract preparation process using enzymatic pretreatment.

Five grams of the defatted powder obtained in Section 2.2.1 were weighed and mixed with 50 mL of a pre-prepared enzymatic solution, consisting of the commercial enzymatic complex Viscozyme^®^ at 2% *v*/*v* in a 0.1 M acetate aqueous buffer at pH 4, based on previous studies [34]. As a control, the same procedure was performed in parallel using 50 mL of the buffer solution without the enzymatic complex. Figure 1 summarizes the extract preparation process using enzymatic pretreatment.

The samples in their respective solutions were sealed under nitrogen and incubated in a G76 thermoregulated water bath (New Brunswick Scientific Co., Edison, NJ, USA) at 37 °C for 16 h. After incubation, solutions were acidified with a 6 M HCl aqueous solution to pH 2 to halt enzymatic activity and freeze-dried to complete dryness. The resulting solid was mixed with 200 mL of a solvent composed of acetone:methanol: water in a 7:7:6 (*v*/*v*/*v*) ratio and incubated in a G76 thermoregulated water bath at 30 °C for 20 min [10]. The mixture was then centrifuged using a Z 206 A centrifuge (HERMLE Labortechnik GmbH, Wehingen, Germany) at 4000× *g* for 5 min, recovering the supernatant and the pellet. This process was repeated two more times using the recovered pellet, and the supernatants were combined. The combined supernatant was concentrated using a rotary evaporator (IKA^®^ HB eco) at 40 °C to remove acetone and methanol, followed by freeze-drying to dryness. The dry extract was resuspended in 5 mL of HPLC-grade methanol, hermetically sealed under a nitrogen atmosphere, and stored at −20 °C. This process resulted in the extracts of soluble phenolics for the Viscozyme and control treatments for both harvest seasons.

To estimate the residual phenolic content after enzyme-assisted extraction, the whole pellet from the previous process (previously weighted to ensure equivalent sample masses, and normalized to the original dry residue mass) was mixed with 50 mL of a 4 M NaOH aqueous solution, hermetically sealed under a nitrogen atmosphere, and incubated for 4 h with magnetic stirring at room temperature. Subsequently, the solution was acidified using a 6 M HCl aqueous solution to pH 2, and five consecutive liquid–liquid extractions were performed, each using 50 mL of ethyl acetate solvent, recovering the organic phase. The organic phase was concentrated using an IKA^®^ HB eco rotary evaporator at 40 °C until dry. The resulting solid was weighed and resuspended in 5 mL of HPLC-grade methanol, hermetically sealed under a nitrogen atmosphere, and stored at −20 °C. This process resulted in the extracts recovered from the insoluble-bound form after Viscozyme pretreatment and the respective control for both harvest seasons.

#### 2.2.3. pH Measurement

The methanol solvent of stored extracts was evaporated, and the dry extracts were reconstituted in distilled water. Then, the pH was measured by inserting the electrode of a previously calibrated pH meter. The measurements were taken three times.

#### 2.2.4. Total Phenolic Content (TPC)

The TPC evaluation was carried out using the Folin–Ciocalteu method, following the protocol described by Oyarzún et al. for microplates [35]. A 10% *v*/*v* solution of Folin–Ciocalteu reagent in distilled water, protected from light, and a 7.5% *w*/*v* sodium carbonate solution in distilled water were prepared. In the microplate, 125 μL of the Folin-Ciocalteu reagent solution was added, followed by 25 μL of the diluted sample, standard solution, or water blank, and finally 100 μL of the sodium carbonate solution. Subsequently, the microplate was incubated for one hour at room temperature in darkness, and absorbance measured using an AMR-100 microplate spectrophotometer (Allsheng Instruments Co., Ltd., Hangzhou, China) at a wavelength of 765 nm. Measurements were done in triplicate. The results were expressed as the mean in mg gallic acid equivalents per 100 g of dry defatted residue.

#### 2.2.5. Identification and Quantification of Phenolic Compounds by UPLC-ESI-MS/MS

Phenolic compounds were identified and quantified following the method described by de Ospina et al. [10] for UPLC-ESI-MS/MS analysis (Ultra-High Performance Liquid Chromatography—Electrospray Ionization—Mass Spectrometry/Mass Spectrometry) [10], with minor modifications, using an ABSciex triple Quad 4500 mass spectrometer equipped with a TurboV electrospray interface coupled to an Eksigent Ekspert Ultra LC100 with an Ekspert Ultra LC100-XL autosampling system (AB/Sciex Concord, ON, Canada). Chromatographic separation of the compounds was carried out using an elution gradient composed of two mobile phases: (A) an aqueous solution of 0.1% *v*/*v* formic acid, and (B) methanol, under the following program: 0 min to 1 min with 15% B, 1 min to 17 min with 15% to 100% B, 17 min to 21 min with 100% B, 21 min to 22 min with 100% to 15% B, and 22 min to 25 min with 15% B. An injection volume of 50 μL, a flow rate of 0.5 mL/min, and a LiChrospher 100 RP-18 column of 125 mm × 4 mm i.d., 5 μm (Merck, Darmstadt, Germany) maintained at 40 °C were used. The identification parameters for each compound are presented in Table 1. Calibration curves were prepared using commercially available standards. The limits of detection (LOD), limit of quantification (LOQ), and r^2^ values for the standards were as follows: gallic acid (LOD = 41 ppb, LOQ = 124 ppb, and r^2^ = 0.9988); caffeic acid (LOD = 142 ppb, LOQ = 430 ppb, and r^2^ = 0.9976); *p*-coumaric acid (LOD = 124 ppb, LOQ = 377 ppb, and r^2^ = 0.9911); ferulic acid (LOD = 110 ppb, LOQ = 334 ppb, and r^2^ = 0.9944); syringic acid (LOD = 55 ppb, LOQ = 167 ppb, and r^2^ = 0.9995); catechin (LOD = 49 ppb, LOQ = 150 ppb, and r^2^ = 0.9997); epicatechin (LOD = 83 ppb, LOQ = 252 ppb, and r^2^ = 0.9991); myricetin (LOD = 180 ppb, LOQ = 545 ppb, and r^2^ = 0.9956); quercetin (LOD = 67 ppb, LOQ = 203 ppb, and r^2^ = 0.9969); and rutin (LOD = 249 ppb, LOQ = 756 ppb, and r^2^ = 0.9939). All compounds were putatively identified with authentic standards using multiple reaction monitoring (Table 1) and retention time (Table S1). Due to an overly-limited amount to obtain a standard curve, results for 3,4-dihydroxybenzoic acid and vanillic acid are expressed as μg equivalents of gallic acid standard per g of dry defatted residue. Results for quercitrin and taxifolin are expressed as μg equivalents of quercetin per g of dry defatted residue. Results for all other detected compounds are expressed as μg of compound per g of dry defatted residue, identified with a standard.

#### 2.2.6. Ferric Reducing Antioxidant Power (FRAP)

The FRAP assay described by Oyarzún et al. for microplates was employed [35]. A 20 mM ferric chloride solution in distilled water, a 40 mM hydrochloric acid solution in water, a 10 mM TPTZ reagent solution in acidic solution, and a 0.3 M acetate buffer solution at pH 3.6 in distilled water were prepared. The FRAP reagent was prepared immediately before microplate filling by mixing the buffer, TPTZ, and ferric chloride solutions in a 10:1:1 volume ratio. On the microplate, 270 μL of FRAP reagent and 30 μL of the diluted sample (in methanol), standard solution, or methanol blank were added. Each well was mixed using a micropipette. The microplate was then incubated for 30 min at room temperature in darkness, protected with aluminum foil, and absorbance was measured using an Allsheng AMR-100 spectrophotometer at a wavelength of 594 nm. Measurements were done in triplicate. Results were expressed as the mean in μmol Trolox equivalents per gram of dry defatted residue.

#### 2.2.7. Oxygen Radical Absorbance Capacity—Fluorescein (ORAC-FL) Method

The ORAC-FL assay was carried out following the method described by Oyarzún et al. for microplates [35]. A 75 mM sodium phosphate-buffered solution at pH 7.4, a 583 nM fluorescein solution in buffer, and a 100 mM AAPH solution in buffer were prepared. Samples were diluted in a buffer solution. On the microplate, 170 μL of buffer solution, 30 μL of fluorescein solution, and 25 μL of the diluted sample, standard solution, or buffer blank were added, and the plate was incubated in the equipment for 20 min at 37 °C. Then, 25 μL of AAPH solution was added, and each well was mixed using a micropipette. The microplate was incubated for 140 min at 37 °C inside a Cytation 5 Cell Imaging Multi-Mode Reader (BioTek Instruments Inc., Winooski, VT, USA), measuring fluorescence every 2 min at an excitation wavelength of 485 nm and an emission wavelength of 528 nm. Fluorescence values obtained were normalized and integrated until fluorescence reached 20% of the maximum for each well. All measurements were done in triplicate. Results were expressed as the mean in μmol Trolox equivalents per gram of dry defatted residue.

#### 2.2.8. ABTS Radical Cation Scavenging

The ABTS method was performed by adapting the protocol described elsewhere [36] for microplate use. A 2.5 mM potassium persulfate solution in water and a 7 mM ABTS stock solution in water were prepared. Both solutions were mixed in a 1:1 volume ratio, and the resulting solution was incubated for 16 h at room temperature in darkness, protected with aluminum foil, to generate the ABTS radical cation. Just before filling the microplate, the ABTS radical cation solution was diluted in methanol to reach a concentration with an absorbance of 0.7 units at 734 nm, monitored with a Thermo Electron Corporation Helios Gamma spectrophotometer. In the microplate, 270 μL of diluted ABTS radical cation solution and 30 μL of the diluted sample (in methanol), standard solution, or methanol blank were added. The microplate was then incubated for 6 min at room temperature in darkness, and absorbance was measured with a ThermoScientific MultiSkan GO spectrophotometer with its software SkanIt RE 7.0.2 at a wavelength of 415 nm. The assay was measured at 415 nm in this microplate adaptation since the colored radical was shown to have an absorbance peak of higher sensitivity at this wavelength in preliminary trials. Measurements were done in triplicate. Results were expressed as the mean in μmol Trolox equivalents per gram of dry defatted residue.

#### 2.2.9. Evaluation of Phenolics Obtained by Enzyme-Assisted Extraction on Lipid Oxidation in Salmon Model System

Ozonized water was used as an oxidation agent for salmon. The ozonation system consisted of an O&L 3.0 RM ozone generator (Ozone&Life, São Paulo, Brazil), an ozonation tower (Ozone&Life, São Paulo, Brazil), an ozone monitor model 106-H (2B Technologies, Boulder, CO, USA), and a thermal catalyst (Ozone&Life, São Paulo, Brazil). A volume of 250 mL of water was placed in the ozonation tower and exposed to an ozone flow of 37 µg/mL for 10 min.

Phenolics obtained by enzyme-assisted extraction (soluble fraction obtained from murtilla pomace subjected to Viscozyme pretreatment, SFVP) were evaluated as a food-grade natural antioxidant for preserving raw salmon, purchased at a local grocery store. Ozone-induced oxidation was performed in a closed system. Briefly, 4 g of fresh salmon were placed into individual tubes (*n* = 3), and treatment solutions were added to a final volume of 1 mL per sample. For comparison purposes with BHT (200 ppm), the total phenolic content (TPC) of both the SFVP extract and the BHT solution was determined. The SFVP extract was rotavaporated and reconstituted to match the same TPC as the BHT treatment. The solutions were added directly onto the salmon tissue and gently mixed to ensure uniform contact between the sample and the reagents.

The samples were divided into the following groups:

- Group 1—Oxidized salmon: 4 g of salmon + 0.5 mL of ozonized water + 0.5 mL of water.
- Group 2—Oxidized salmon + SFVP: 4 g of salmon + 0.5 mL of ozonized water + 0.5 mL of SFVP 200 ppm gallic acid equivalent as measured considering its TPC.
- Group 3—Oxidized salmon + BHT 200 ppm: 4 g of salmon + 0.5 mL of ozonized water + 0.5 mL of BHT.
- Group 4—Control: 4 g of salmon + 1 mL of water.

All Salmon groups were stored at 4 °C, and lipid oxidation was assessed on days 0, 2, and 4, using the thiobarbituric acid reactive substances (TBARS) assay and analyzed using the thiobarbituric acid reactive substances (TBARs) assay. Briefly, 1 g of frozen raw salmon samples was mixed with 2.5 mL of trichloroacetic acid (10% *w*/*v*) and vortexed for 2 min. Next, it was combined with 2.5 mL of aqueous 2-thiobarbituric acid solution (0.02 M), vortexed for 30 s, and centrifuged at 3000× *g* for 10 min. The supernatant was recovered and heated in boiling water for 45 min. Finally, the sample was cooled, 250 uL were collected, and absorbance was measured at 532 nm using a microplate reader spectrophotometer (Synergy HTX S1LFA-SN, Biotek, VT, USA). A standard malondialdehyde (MDA) curve was prepared within the 1-10 ppm concentration range, using 1,1,3,3-Tetraethoxypropane as the MDA precursor. The TBARS values were expressed as MDA equivalents per kilogram of salmon (mg MDA eq/kg).

### 2.3. Statistical Analysis

Data were analyzed using Student’s *t*-distribution to compare two sets of data, and one-way ANOVA followed by Tuckey’s range test to compare more than two sets of data when needed. A difference in data sets was considered statistically significant when *p* < 0.05 for all tests. Data analysis was performed via GraphPad PRISM 8.0.1 software. For all data obtained by algebraic operations, standard deviation was calculated using the relevant formulae for the principle of propagation of uncertainty.

## 3. Results

### 3.1. Enzymatic Pretreatment Increases Total Phenolics and Antioxidant Activity in the Soluble Fraction

The pH of all concentrated extracts were in the range of 2.40 ± 0.12 units. Data obtained for TPC and antioxidant activity measured by different methods are presented in Table 2. Results for TPC of the soluble fraction demonstrate that the applied enzymatic treatment was effective in releasing up to 57% more phenolic compounds compared to the control. This indicates that enzymes in the Viscozyme enzymatic mixture were able to break some of the bonds linking the phenolic compounds to the cell wall fibers in the murtilla juice residues. Moreover, the phenolic hydrolysates released from the insoluble-bound form obtained through an alkaline hydrolysis showed a lower concentration of phenolic compounds compared to the control (untreated with Viscozyme). This supports the idea that a significant amount of phenolic compounds had already been released during the initial enzymatic step, leaving fewer compounds for alkaline hydrolysis to release.

The FRAP assay indicated that in the soluble fraction, treatment with Viscozyme produced an extract with higher reducing power than its respective control. This method quantifies only the antioxidant activity mediated by single electron transfer (SET) mechanisms [37], so any antioxidant activity mediated by hydrogen atom transfer (HAT) mechanisms is not reflected in this assay. These results are consistent with the increase in total phenolic content, suggesting that the compounds released during enzymatic pretreatment contribute significantly to the reducing capacity of the extract.

Evaluation of antioxidant capacity using the ORAC-FL assay indicated that the soluble fraction recovered from samples treated with Viscozyme exhibited higher antioxidant capacity than its control. In the insoluble-bound fraction, this assay indicated that samples treated with Viscozyme exhibited lower antioxidant capacity, in line with the TPC, suggesting that enzymatic action released the compounds during the first extraction step by the action of Viscozyme. This assay is valued as an approach to biological systems since it uses peroxyl radicals to oxidize the fluorescent probe, which more accurately simulates the reality in a wide variety of biological matrices, providing greater weight to the interpretation of physiologically relevant antioxidant activity [38]. Similarly, the higher ORAC-FL values observed in the soluble fraction further support the increase in phenolic compounds and indicate an enhanced capacity to neutralize peroxyl radicals, which are key species involved in lipid oxidation processes.

The ABTS method, like the FRAP assay, showed an increase in antioxidant activity in the soluble fraction treated with Viscozyme compared to its control and no significant difference in the insoluble-bound fraction. This assay, however, uses complex free radicals with a large number of structures near the nitrogen atom carrying the unpaired electron, which is thought to introduce steric hindrance that limits its reactivity [39]. The ABTS radical used can be reduced through both single electron transfer (SET) and hydrogen atom transfer (HAT) mechanisms, complementing the information provided by the previous assays [40].

Overall, the agreement between TPC and the different antioxidant assays reinforces the effectiveness of enzymatic pretreatment in enhancing the release of phenolic compounds in the soluble fraction. Although it is well established that correlations between chemical-based antioxidant assays and oxidative stability in complex food matrices are not always linear, highlighting the importance of validating antioxidant activity directed in the target system.

### 3.2. Enzyme Pretreatment Changes the Phenolic Profile in the Soluble and Insoluble-Bound Fraction

Table 3 presents the results obtained by UPLC-ESI-MS/MS, where the soluble and insoluble-bound phenolics, both from enzymatic pretreatment and the control, were analyzed, as these samples exhibited the highest concentration of TPC. Seven phenolic acids and seven flavonoids were detected and quantified. In the soluble fraction, the compound detected at the highest concentration level in the sample subjected to enzymatic pretreatment was quercetin, while in the control extract, it was rutin, a quercetin glycoside. In the extract containing phenolics released from the insoluble-bound form, the compound present in the highest concentration was gallic acid for both the enzymatic and control extracts.

The UPLC-ESI-MS/MS quantification of the extracts revealed differences in the phenolic compound profile when comparing the extracts from the soluble fraction. In the enzymatic pre-treatment extract, an increase was observed in most of the phenolic acids (syringic, vanillic, 3,4-dihydroxybenzoic, caffeic, p-coumaric, and ferulic acids) and flavonoids (quercetin, myricetin, quercitrin, catechin, epicatechin, and taxifolin) compared to the control extract. This aligns with the results for TPC presented earlier for these extracts, where both parameters were found to be higher in samples subjected to enzymatic pretreatment compared to the control. The compounds most positively affected were ferulic acid (+314.6%), caffeic acid (+249.4%), taxifolin (+186.8%), 3,4-dihydroxybenzoic acid (+129.6%), and p-coumaric acid (+119.6%). However, it is important to note that in the control, the major compounds were rutin, followed by quercetin and gallic acid. Conversely, in enzymatically treated samples, the major compounds were quercetin, followed by gallic acid and quercitrin.

In the phenolic extract obtained from the insoluble-bound form enzymatic pretreatment translated into a lower concentration of most detected compounds (gallic acid, syringic acid, vanillic acid, dihydroxybenzoic acid, caffeic acid, p-coumaric acid, ferulic acid, quercitrin, epicatechin, and taxifolin) was observed, consistent with the data obtained for TPC in the insoluble-bound fraction analyzed earlier. The concentration of catechin was not significantly affected between the two extracts, and its concentration was considerably higher in both extracts of the insoluble-bound form than in the extracts of the soluble fraction. This suggests that enzymatic action releases a certain proportion of catechin bound to the cell wall fibers, but this proportion does not create a significant difference in the total amount of catechin remaining bound to those fibers. Finally, a higher concentration of quercetin and myricetin in the enzymatic extract of the insoluble-bound form could be attributed to the fact that these compounds may be present in forms more accessible to enzymatic action, and therefore be preferentially released during enzymatic treatment, possibly because the enzymes may break bonds that hold these molecules bound to fibers less accessible to alkaline hydrolysis. This suggests that while alkaline hydrolysis is more effective at releasing a wider variety of phenolic compounds, enzymatic treatment can be selective and preferentially release certain compounds depending on how they are linked to the cellular matrices in murtilla.

### 3.3. Soluble Phenolics of Murtilla Pomace Subjected to Viscozyme Pretreatment Delay Lipid Oxidation Against Ozone-Induced Oxidation in a Salmon Model System

To address the relevance in the food matrix, we complemented chemical-based assays with lipid peroxidation measurements in fresh salmon samples. TBARS (thiobarbituric acid reactive substances) indicate secondary lipid oxidation and have been widely used in shelf-life studies. Table 4 shows the oxidative stability of salmon during storage under refrigeration. The oxidation level increased 4.3 times in samples that did not receive any antioxidant (control). In contrast, soluble phenolics of murtilla pomace subjected to Viscozyme pretreatment delayed the oxidation during storage, decreasing this level by up to 67.8% on day 4. Interestingly, no differences were found between the effect of this extract, which contains natural antioxidants, and BHT, a commonly used synthetic antioxidant, on extending the shelf life of foods.

This preservative effect can be attributed to the phenolic compounds present in the extract, which act as chain-breaking antioxidants by scavenging free radicals and interrupting lipid peroxidation. The increase in total phenolic content after enzymatic pretreatment likely enhanced this effect, contributing to the reduction in oxidation observed by TBARS in the salmon system. These results provide direct evidence of the potential protective effect of the soluble fraction extract under real conditions.

## 4. Discussion

Out of all compounds identified in the samples, the three found in the highest concentration were gallic acid, quercetin and rutin, three phenolics that have been widely studied for their health benefits. Gallic acid is notable for its antioxidant, anti-inflammatory, and antineoplastic activities, as well as its therapeutic effects on gastrointestinal, neuropsychological, metabolic, and cardiovascular diseases [41]. Quercetin, in turn, is recognized for its antioxidant, anti-inflammatory, antiproliferative, anticancer, antidiabetic, and antiviral properties, as well as its ability to cross the blood–brain barrier, making it a potential protective agent against neurodegenerative diseases [42]. Finally, rutin stands out for its antioxidant, anti-inflammatory, antimicrobial, and antidiabetic properties [43]. The significant biological relevance of the major compounds found in the extracts supports the potential value of recovering polyphenols from the residue generated during murtilla juice production. Since murtilla juice pomace is usually discarded as waste [10], there is an economic potential for the recovery of any compound useful for the industry. While the commercial relevance of these findings remains to be established, as it falls beyond the scope of the present study, the total phenolic content (TPC) of murtilla pomace (1600.6–1610.1 mg GAE/100 g dry residue) can be compared with that of apple pomace (555–1376 mg GAE/100 g dry residue) [44], which is already of commercial importance.

The TPC of the soluble fraction of the residue obtained through enzymatic treatment was approximately 46% of the TPC for intact murtilla fruit. This indicates that the residue from murtilla juice production has the potential to be valorized as an important source of phenolic compounds. However, it is important to note that all values obtained for concentrations of the evaluated compounds in the soluble fraction are considerably lower than those reported in previous studies on murtilla fruit [45]. For instance, the literature indicates that each gram of dried murtilla fruit contains 410 µg of gallic acid, 467 µg of quercetin, and 817 µg of rutin [46]. This suggests that during the juice production process, some of the phenolic compounds in the fruit were released from the mashed cells into the juice, and as such did not remain in the residue, explaining the significant differences in compound levels compared to the literature.

Several studies conducted on other plant matrices have reported an increase in the concentration of phenolic compounds after applying a treatment with the Viscozyme enzymatic mixture [47,48,49,50]. A higher concentration of rutin in the control extract compared to the enzymatic extract is consistent with previous studies showing that the employed enzymes are capable of biotransforming this compound [51,52], releasing the aglycone quercetin and the sugar rutinose. This explains why rutin is significantly reduced in the enzymatically treated extract. It is worth mentioning that quercitrin, another quercetin glycoside identified in the samples, did not exhibit the same behavior. This difference may be due to the presence of a specific enzyme in the mixture for the hydrolysis of rutin, which does not affect other quercetin glycosides.

Antioxidants play a crucial role in mitigating oxidative stress and, therefore, preventing lipid oxidation. Although correlations between chemical-based antioxidant assays and oxidative stability in complex food matrices are not always linear, highlighting the importance of validating antioxidant activity directly in the target system [53]. In vitro methods to assess antioxidant capacity, such as the ORAC assay, measure the chain-breaking ability of antioxidants against free radicals, particularly peroxyl radicals, which are highly relevant in lipid oxidation processes [54]. Furthermore, the effectiveness of phenolic compounds is strongly influenced by their polarity, which determines their partitioning and localization within the fish matrix. In lipid-based systems, oxidation is initiated at interfacial regions; for instance, antioxidants properly localized at these sites can more effectively inhibit lipid peroxidation [55].

Additionally, a significant correlation between sensory evaluation and TBARS values has been reported, supporting the use of TBARS as a reliable indicator of lipid oxidation and shelf-life in fish products [56]. The preservation of fish quality during storage is a critical concern in the food industry due to its susceptibility to oxidative deterioration [57]. For instance, BHT is one of the most used synthetic antioxidants. However, there has been a concern regarding its safety [58]. The preservative effect of soluble phenolics of murtilla pomace subjected to Viscozyme pretreatment demonstrates its potential to decrease oxidative reactions and the formation of undesirable chemical products. Some authors have reported the positive effect of phenolic extracts from natural sources in delaying the increase in oxidation products [59]. However, the present study is the first to evaluate the effect of murtilla pomace subjected to Viscozyme pretreatment, especially when it comes to the effect in an ozone-induced oxidation in a salmon model. Therefore, comparison may be difficult. The phenolic extract used in the present study contains phenolic acids and flavonoids, mostly quercetin and its derivatives. Other authors found that a superficial treatment with dihydroquercitin makes hydrolytic and oxidative changes in fish lipids during storage for up to eleven days, in contrast with our study, which recorded a preservation of up to 4 days of storage under refrigeration [60].

## 5. Conclusions

Our previous study demonstrated that more than half of the total phenolic compounds present in the residue from murtilla juice, namely murtilla pomace, are in the insoluble-bound form [10], demonstrating that murtilla pomace, such as the fruit, is a great source of natural antioxidants. In this study, the effect of Viscozyme-assisted extraction on phenolic recovery from murtilla pomace was addressed. Total phenolic content in the soluble fraction was 1.5 times higher in the extracts obtained through enzymatic pretreatment compared to the control extracts. The main compounds in the control extracts were rutin, followed by quercetin and gallic acid. In contrast, in samples subjected to enzymatic pretreatment, the main compounds were quercetin, followed by gallic acid and quercitrin. The antioxidant potential was positively affected by enzymatic pretreatment. Soluble phenolics of murtilla pomace subjected to Viscozyme pretreatment showed antioxidant properties in ozone-induced oxidation using salmon as a model system. In summary, extraction assisted by pretreatment with Viscozyme led to the release of soluble phenolics present in the residue from murtilla juice production (murtilla pomace), enhancing their antioxidant potential and effectively delaying lipid oxidation in the salmon model system, showing comparable performance to the synthetic antioxidant BHT. These findings highlight the potential of this extract as a natural alternative for controlling oxidation in salmon systems. Furthermore, these results provide a basis for future studies aimed at complementing these findings with additional evaluations, including volatile compounds related to salmon oxidation, to better understand scalability in an industrial environment and seek authorization from regulatory agencies for its commercial use.

## Figures and Tables

**Table 1:** UPLC-ESI-MS/MS identification of phenolic compounds in murtilla pomace.

Compound	MRM Transition 1	DP	CE	CXP	MRM Transition 2	DP	CE	CXP
3,4-Dihydroxybenzoic acid	152.0 > 107.9	−50	−26	−3	152.9 > 108.9	−50	−16	−3
Gallic acid	168.9 > 124.9	−70	−18	−15	168.9 > 78.9	−70	−28	−15
*p*-coumaric acid	162.9 > 119.0	−70	−20	−5	162.9 > 119.0	−70	−38	−25
Vanillic acid	166.9 > 120.8	−5	−12	−11	166.9 > 151.8	−5	−16	−13
Caffeic acid	178.9 > 135.0	−70	−20	−5	178.9 > 133.9	−70	−32	−7
Ferulic acid	193.0 > 134.0	−55	−20	−7	193.0 > 177.9	−55	−16	−15
Syringic acid	197.0 > 181.9	−65	−18	−5	197.0 > 122.9	−65	−30	−7
Catechin	289.0 > 24.0	−100	−22	−13	289.0 > 108.9	−100	−30	−7
Epicatechin	289.0 > 244.9	−110	−20	−19	289.0 > 109.0	−110	−30	−7
Quercetin	301.0 > 150.9	−15	−28	−13	301.0 > 178.8	−15	−24	−11
Miricetin	316.9 > 150.9	−105	−30	−7	316.9 > 178.9	−105	−26	−9
Quercitrin	447.0 > 299.9	−105	−32	−13	447.0 > 299.9	−105	−30	−7
Taxifolin	302.9 > 285.0	−105	−14	−5	302.9 > 125.0	−105	−30	−7
Rutin	609.0 > 299.8	−170	−50	−13	609.0 > 300.5	−170	−42	−9

Abbreviations: MRM = multiple reaction monitoring; DP = declustering potential; CE = collision energy; CXP = collision cell exit potential.

**Table 2:** Effect of enzyme pretreatment on recovery of phenolics from murtilla pomace.

		Soluble Phenolics (SPs)	Insoluble-Bound Phenolics (IBPs)	Proportion (SPs/IBPs)
**Total Phenolics (mg GAE/100 g DDR)**				
2022 Harvest	Viscozyme^®^	1610.1 ± 84.6 **	2018.7 ± 95.6 **	0.80 ± 0.06 ***
	Control	1025.3 ± 73.2	2336.5 ± 50.7	0.44 ± 0.03
2023 Harvest	Viscozyme^®^	1600.6 ± 63.5 ***	2053.9 ± 65.6 **	0.78 ± 0.04 ***
	Control	1039.6 ± 63.6	2303.0 ± 40.5	0.45 ± 0.03
**FRAP Antioxidant Activity (μmol TE/g DDR)**				
2022 Harvest	Viscozyme^®^	108.9 ± 14.5 **	208.6 ± 20.5 ^ns^	0.52 ± 0.09 ^ns^
	Control	90.0 ± 12.4	216.2 ± 21.0	0.42 ± 0.07
2023 Harvest	Viscozyme^®^	116.2 ± 13.0 **	219.7 ± 22.5 ^ns^	0.53 ± 0.08 ^ns^
	Control	85.5 ± 12.0	213.1 ± 18.3	0.40 ± 0.07
**ORAC-FL Antioxidant Activity (μmol TE/g DDR)**				
2022 Harvest	Viscozyme^®^	134.7 ± 4.7 **	532.3 ± 5.2 *	0.25 ± 0.01 *
	Control	117.6 ± 4.8	628.5 ± 5.8	0.19 ± 0.01
2023 Harvest	Viscozyme^®^	133.6 ± 1.4 **	536.1 ± 1.4 *	0.21 ± 0.00 *
	Control	119.4 ± 1.7	624.1 ± 2.1	0.19 ± 0.00
**ABTS Antioxidant Activity (μmol TE/g DDR)**				
2022 Harvest	Viscozyme^®^	115.8 ± 11.3 ***	210.8 ± 9.4 ^ns^	0.55 ± 0.06 **
	Control	84.8 ± 12.4	232.1 ± 8.2	0.36 ± 0.05
2023 Harvest	Viscozyme^®^	119.7 ± 9.8 ***	216.3 ± 7.6 ^ns^	0.55 ± 0.05 **
	Control	87.9 ± 10.4	227.2 ± 7.7	0.39 ± 0.05

Data shown represents the mean ± standard deviation of three independent experiments for each assay, and the proportion shown corresponds to the division of relevant values ± calculated error propagation. Symbols represent statistical differences with respect to control in the same column and the same harvest year. Abbreviations: GAE = gallic acid equivalents; TE = Trolox equivalents; DDR = defatted dry residue; ns = non significant, *p* > 0.05; * = *p* < 0.05; ** = *p* < 0.01; *** = *p* < 0.001.

**Table 3:** Effect of enzymatic pretreatment on either soluble-bound or insoluble-bound phenolics (μg/g DDR) from murtilla pomace as evaluated by UPLC-ESI-MS/MS.

	Soluble Phenolics (SPs)			Insoluble-Bound Phenolics (IBPs)		
Phenolic Compound ^a^	Viscozyme^®^	Control	Variation (%)	Viscozyme^®^	Control	Variation (%)
**Phenolic Acids**						
Gallic acid	101.55 ± 9.34 ^ns^	96.43 ± 3.4	*ns*	881.05 ± 17.22 *	973.66 ± 42.26	−9.5%
Syringic acid	0.72 ± 0.01 ****	0.47 ± 0.00	+53.2%	0.69 ± 0.01 ****	0.84 ± 0.01	−17.8%
Vanillic acid	1.59 ± 0.08 ****	0.93 ± 0.60	+71.0%	0.89 ± 0.29 ***	1.32 ± 0.15	−32.6%
3,4-dihydroxybenzoic acid	2.71 ± 0.01 ****	1.18 ± 0.00	+129.6%	17.42 ± 0.41 ***	23.05 ± 0.55	−24.4%
Caffeic acid	2.83 ± 0.07 ****	0.81 ± 0.02	+249.4%	4.69 ± 0.16 ***	5.83 ± 0.10	−19.5%
*p*-coumaric acid	42.48 ± 0.55 ****	19.34 ± 0.09	+119.6%	20.32 ± 0.29 ****	30.53 ± 0.62	−33.4%
Ferulic acid	4.81 ± 0.17 ****	1.16 ± 0.04	+314.6%	15.69 ± 0.06 ****	20.34 ± 0.18	−22.9%
**Flavonoids**						
Quercetin	214.04 ± 4.83 ****	127.97 ± 3.33	+67.2%	85.50 ± 2.39 ***	61.57 ± 2.55	+38.9%
Miricetin	28.09 ± 0.44 ****	17.05 ± 0.43	+64.7%	21.35 ± 0.31 **	19.73 ± 0.12	+8.2%
Rutin	4.66 ± 0.48 ****	158.78 ± 0.85	−97.1%	*tr*	*tr*	*ns*
Quercitrin	47.80 ± 0.66 ***	43.08 ± 0.64	+10.9%	3.74 ± 0.01 ****	5.72 ± 0.06	−34.6%
Catechin	9.66 ± 0.24 ***	8.35 ± 0.07	+15.7%	76.89 ± 0.99 ^ns^	77.65 ± 0.63	*ns*
Epicatechin	4.83 ± 0.08 *	4.34 ± 0.21	+11.3%	37.72 ± 0.94 *	39.77 ± 0.59	−5.1%
Taxifolin	37.31 ± 0.49 ****	13.01 ± 0.25	+186.8%	15.95 ± 0.05 ****	25.97 ± 0.24	−38.6%
**Phenolic Acids Sum**	156.69 ± 10.23 **	120.32 ± 4.15	+30.2%	940.75 ± 18.44 *	1055.57 ± 43.87	−10.9%
**Flavonoids Sum**	346.39 ± 7.22 **	372.58 ± 5.78	−7.0%	241.15 ± 4.69 *	230.41 ± 4.19	+4.7%

^a^ Concentrations are expressed in μg of compound per g defatted dry residue (DDR). Concentrations of 3,4-dihydroxybenzoic acid and vanillic acid are expressed in μg gallic acid equivalents per g DDR. Concentrations of quercitrin and taxifolin are expressed in μg quercetin equivalents per g DDR. Data shown represents mean ± standard deviation of three independent measurements, or the sum of relevant values ± calculated error propagation. Control corresponds to treatment with acetate buffer 0.1 M pH 4 instead of 2% Viscozyme^®^ solution in the same buffer. The increase was calculated with respect to the control. Symbology: *tr* = trace; *ns* = non-significant, *p* > 0.05; * = *p* < 0.05; ** = *p* < 0.01; *** = *p* < 0.001; **** = *p* < 0.0001.

**Table 4:** Effect of soluble phenolics of murtilla pomace subjected to enzymatic pretreatment against ozone-induced oxidation in a salmon model system.

Salmon Sample	mg MDA eq/kg		
	Day 0	Day 2	Day 4
Ozone	4.95 ± 1.34	20.52 ± 1.58 ^a^	21.31 ± 7.39 ^a^
Ozone + BHT	3.94 ± 1.15	16.90 ± 0.65 ^b^	8.38 ± 2.68 ^b^
Ozone + SFVP	5.69 ± 0.83	15.81 ± 0.52 ^b^	6.86 ± 3.49 ^b^

Data shown represents the mean ± standard deviation of three independent measurements. Statistical analysis with one-way ANOVA between groups, post hoc Tukey; different letters in the columns imply statistical differences, *p* < 0.05 among treatments. Abbreviations: BHT = butylhydroxytoluene 200 ppm; SFVP = soluble fraction obtained from murtilla pomace subjected to Viscozyme pre-treatment (200 ppm gallic acid equivalent as measured considering its total phenolic content); MDA eq = malonaldehyde equivalent.

## Data Availability

The original contributions presented in this study are included in the article. Further inquiries can be directed to the corresponding authors.

## Supplementary Materials

The following supporting information can be downloaded at https://www.mdpi.com/article/10.3390/antiox15050593/s1. Table S1: UPLC-ESI-MS/MS representative chromatograms of phenolic compounds in murtilla juice residue.

## Abbreviations

The following abbreviations are used in this manuscript:

TPC	Total phenolic content
DPPH	2,2-diphenyl-1-picrylhydrazyl
ABTS	2,2′-azino-bis(3-ethylbenzothiazoline-6-sulfonic) acid
AAPH	2,2-azobis(2-amidinopropane) dihydrochloride
TPTZ	2,4,6-tris(2-pyridyl)1,3,5-triazine
UPLC-ESI-MS/MS	Ultra-High Performance Liquid Chromatography—Electrospray Ionization—Mass Spectrometry/Mass Spectrometry
HPLC	High Performance Liquid Chromatography
MRM	Multiple Reaction Monitoring.
DP	Declustering Potential
CE	Collision Energy
CXP	Collision cell exit Potential
FRAP	Ferric Reducing Antioxidant Power
ORAC-Fl	Oxygen Radical Absorbance Capacity–Fluorescein
SFVP	Soluble Fraction obtained from murtilla pomace subjected to Viscozyme Pretreatment
BHT	Butylhydroxytoluene
TBARs	Thiobarbituric Acid Reactive substances
MDA	Malondialdehyde
SET	Single Electron Transfer
HAT	Hydrogen Atom Transfer
SP	Soluble Phenolics
IBP	Insoluble-Bound Phenolics
GAE	Gallic Acid Equivalents
TE	Trolox Equivalents
DDR	Defatted Dry Residue
*ns*	Non-significant
*tr*	Trace amounts

## References

[1] Speisky H., López-Alarcón C., Gómez M., Fuentes J., Sandoval-Acuña C. (2012). First Web-Based Database on Total Phenolics and Oxygen Radical Absorbance Capacity (ORAC) of Fruits Produced and Consumed within the South Andes Region of South America. J. Agric. Food Chem..

[2] Espinoza-Tellez T., Bastías-Montes J.M., Quevedo-León R., Valencia-Aguilar E., Díaz-Carrasco O., Solano-Cornejo M.Á., Mesa-Mesina F. (2021). La murta (*Ugni molinae*) y sus propiedades benéficas para la salud: Una revisión. Sci. Agropecu..

[3] Shene C., Reyes A.K., Villarroel M., Sineiro J., Pinelo M., Rubilar M. (2009). Plant location and extraction procedure strongly alter the antimicrobial activity of murta extracts. Eur. Food Res. Technol..

[4] Schreckinger M.E., Lotton J., Lila M.A., de Mejia E.G. (2010). Berries from South America: A Comprehensive Review on Chemistry, Health Potential, and Commercialization. J. Med. Food.

[5] Delporte C., Backhouse N., Inostroza V., Aguirre M.C., Peredo N., Silva X., Negrete R., Miranda H.F. (2007). Analgesic activity of *Ugni molinae* (murtilla) in mice models of acute pain. J. Ethnopharmacol..

[6] Arancibia-Radich J., González-Blázquez R., Alcalá M., Martín-Ramos M., Viana M., Arribas S., Delporte C., Fernández-Alfonso M.S., Somoza B., Gil-Ortega M. (2019). Beneficial effects of murtilla extract and madecassic acid on insulin sensitivity and endothelial function in a model of diet-induced obesity. Sci. Rep..

[7] Suwalsky M., Orellana P., Avello M., Villena F. (2007). Protective effect of *Ugni molinae* Turcz. against oxidative damage of human erythrocytes. Food Chem. Toxicol..

[8] Aguirre M.C., Delporte C., Backhouse N., Erazo S., Letelier M.E., Cassels B.K., Silva X., Alegría S., Negrete R. (2006). Topical anti-inflammatory activity of 2α-hydroxy pentacyclic triterpene acids from the leaves of *Ugni molinae*. Bioorg. Med. Chem..

[9] Karoney E.M., Molelekoa T., Bill M., Siyoum N., Korsten L. (2024). Global research network analysis of fresh produce postharvest technology: Innovative trends for loss reduction. Postharvest Biol. Technol..

[10] Ospina-Posada A.C., Porras O., Rincón-Cervera M.A., Frias J., Zielinski A.A.F., Bridi R., Arias-Santé M.F., de Camargo A.C. (2024). Antioxidant properties of phenolic extracts of murtilla pomace: First report on the importance of soluble and insoluble-bound compounds. Food Res. Int..

[11] Osorio L.L.D.R., Flórez-López E., Grande-Tovar C.D. (2021). The Potential of Selected Agri-Food Loss and Waste to Contribute to a Circular Economy: Applications in the Food, Cosmetic and Pharmaceutical Industries. Molecules.

[12] Sreekala A.G.V., Ismail M.H.B., Nathan V.K. (2022). Biotechnological interventions in food waste treatment for obtaining value-added compounds to combat pollution. Environ. Sci. Pollut. Res..

[13] Asif M., Javaid T., Razzaq Z.U., Khan M.K.I., Maan A.A., Yousaf S., Usman A., Shahid S. (2024). Sustainable utilization of apple pomace and its emerging potential for development of functional foods. Environ. Sci. Pollut. Res..

[14] Hasan M.M., Islam M.R., Haque A.R., Kabir M.R., Khushe K.J., Hasan S.M.K. (2024). Trends and challenges of fruit by-products utilization: Insights into safety, sensory, and benefits of the use for the development of innovative healthy food: A review. Bioresour. Bioprocess..

[15] Pedro A.C., Maciel G.M., Lima N.P., Lima N.F., Ribeiro I.S., Pinheiro D.F., Haminiuk C.W.I. (2024). Valorization of bioactive compounds from juice industry waste: Applications, challenges, and future prospects. Trends Food Sci. Technol..

[16] Yang K., Deng X., Jian S., Zhang M., Wen C., Xin Z., Zhang L., Tong A., Ye S., Liao P. (2022). Gallic Acid Alleviates Gut Dysfunction and Boosts Immune and Antioxidant Activities in Puppies Under Environmental Stress Based on Microbiome–Metabolomics Analysis. Front. Immunol..

[17] Bianchi S., Marchese P., Vannini M., Sisti L., Tassoni A., Ferri M., Mallegni N., Cinelli P., Celli A. (2023). Evaluation of the activity of natural phenolic antioxidants, extracted from industrial coffee residues, on the stability of poly(1,4-butylene succinate) formulations. J. Appl. Polym. Sci..

[18] El-Sayed S.M., Shazly A.B. (2024). Merging the spring onion extract into soft cheese as a rich natural phenolic ingredient to improve its antioxidant, functional, and sensory properties. J. Food Meas. Charact..

[19] Kumar N., Goel N. (2019). Phenolic acids: Natural versatile molecules with promising therapeutic applications. Biotechnol. Rep..

[20] Lourenço S.C., Moldão-Martins M., Alves V.D. (2019). Antioxidants of Natural Plant Origins: From Sources to Food Industry Applications. Molecules.

[21] Mahfuz S., Shang Q., Piao X. (2021). Phenolic compounds as natural feed additives in poultry and swine diets: A review. J. Anim. Sci. Biotechnol..

[22] Mohd Azman N.A., Gallego M.G., Segovia F., Abdullah S., Shaarani S.M., Almajano Pablos M.P. (2016). Study of the Properties of Bearberry Leaf Extract as a Natural Antioxidant in Model Foods. Antioxidants.

[23] Villalobos M.d.C., Serradilla M.J., Martín A., Ordiales E., Ruiz-Moyano S., Córdoba M.d.G. (2016). Antioxidant and antimicrobial activity of natural phenolic extract from defatted soybean flour by-product for stone fruit postharvest application. J. Sci. Food Agric..

[24] Rasera G.B., Bridi R., Danielski R., Shahidi F., de Camargo A.C. (2024). Phenolic antioxidants in the framework of Sustainable Development Goals: How far are we from zero waste?. Curr. Opin. Food Sci..

[25] Gligor O., Mocan A., Moldovan C., Locatelli M., Crișan G., Ferreira I.C.F.R. (2019). Enzyme-assisted extractions of polyphenols—A comprehensive review. Trends Food Sci. Technol..

[26] Li Z., Smith K.H., Stevens G.W. (2016). The use of environmentally sustainable bio-derived solvents in solvent extraction applications—A review. Chin. J. Chem. Eng..

[27] Soquetta M.B., Terra Lde M., Bastos C.P. (2018). Green technologies for the extraction of bioactive compounds in fruits and vegetables. CyTA—J. Food.

[28] Kim Y., Oh J., Jang C.H., Lim J.S., Lee J.S., Kim J.-S. (2020). In Vivo Anti-Inflammatory Potential of Viscozyme^®^-Treated Jujube Fruit. Foods.

[29] Zhang J., Li M., Cheng J., Zhang X., Li K., Li B., Wang C., Liu X. (2021). Viscozyme L hydrolysis and Lactobacillus fermentation increase the phenolic compound content and antioxidant properties of aqueous solutions of quinoa pretreated by steaming with α-amylase. J. Food Sci..

[30] Jagelaviciute J., Staniulyte G., Cizeikiene D., Basinskiene L. (2023). Influence of Enzymatic Hydrolysis on Composition and Technological Properties of Apple Pomace and Its Application for Wheat Bread Making. Plant Foods Hum. Nutr..

[31] Radenkovs V. (2013). Wheat Bran Carbohydrates as Substrate for Bifidobacterium lactis Development. Int. J. Biol. Biomol. Agric. Food Biotechnol. Eng..

[32] Pinto de Rezende L., Barbosa J., Teixeira P. (2022). Analysis of alternative shelf life-extending protocols and their effect on the preservation of seafood products. Foods.

[33] Food and Agriculture Organization of the United Nations. FAO (2023). Salmon—Main Producers See Record-Breaking Exports. https://www.fao.org/in-action/globefish/news-events/news/news-detail/Salmon---Main-producers-see-record-breaking-exports.

[34] de Camargo A.C., Regitano-d’Arce M.A.B., Biasoto A.C.T., Shahidi F. (2014). Enzyme-assisted extraction of phenolics from winemaking by-products: Antioxidant potential and inhibition of alpha-glucosidase and lipase activities. Food Chem..

[35] Oyarzún J.E., Andia M.E., Uribe S., Núñez Pizarro P., Núñez G., Montenegro G., Bridi R. (2021). Honeybee Pollen Extracts Reduce Oxidative Stress and Steatosis in Hepatic Cells. Molecules.

[36] de Camargo A.C., Vieira T.M.F.d.S., Regitano-D’Arce M.A.B., Calori-Domingues M.A., Canniatti-Brazaca S.G. (2012). Gamma Radiation Effects on Peanut Skin Antioxidants. Int. J. Mol. Sci..

[37] Prior R.L., Wu X., Schaich K. (2005). Standardized Methods for the Determination of Antioxidant Capacity and Phenolics in Foods and Dietary Supplements. J. Agric. Food Chem..

[38] Zhong Y., Shahidi F., Shahidi F. (2015). Methods for the assessment of antioxidant activity in foods. Handbook of Antioxidants for Food Preservation.

[39] Bibi Sadeer N., Montesano D., Albrizio S., Zengin G., Mahomoodally M.F. (2020). The Versatility of Antioxidant Assays in Food Science and Safety—Chemistry, Applications, Strengths, and Limitations. Antioxidants.

[40] Apak R., Özyürek M., Güçlü K., Çapanoğlu E. (2016). Antioxidant Activity/Capacity Measurement. 2. Hydrogen Atom Transfer (HAT)-Based, Mixed-Mode (Electron Transfer (ET)/HAT), and Lipid Peroxidation Assays. J. Agric. Food Chem..

[41] Kahkeshani N., Farzaei F., Fotouhi M., Alavi S.S., Bahramsoltani R., Naseri R., Momtaz S., Abbasabadi Z., Rahimi R., Farzaei M.H. (2019). Pharmacological effects of gallic acid in health and disease: A mechanistic review. Iran. J. Basic Med. Sci..

[42] Deepika, Maurya P.K. (2022). Health Benefits of Quercetin in Age-Related Diseases. Molecules.

[43] Semwal R., Joshi S.K., Semwal R.B., Semwal D.K. (2021). Health benefits and limitations of rutin—A natural flavonoid with high nutraceutical value. Phytochem. Lett..

[44] Li W., Yang R., Ying D., Yu J., Sanguansri L., Augustin M.A. (2020). Analysis of polyphenols in apple pomace: A comparative study of different extraction and hydrolysis procedures. Ind. Crops Prod..

[45] López de Dicastillo C., Bustos F., Valenzuela X., López-Carballo G., Vilariño J.M., Galotto M.J. (2017). Chilean berry *Ugni molinae* Turcz. Fruit and leaves extracts with interesting antioxidant, antimicrobial and tyrosinase inhibitory properties. Food Res. Int..

[46] Gómez-Pérez L.S., Moraga N., Ah-Hen K.S., Rodríguez A., Vega-Gálvez A. (2022). Dietary fibre in processed murta (*Ugni molinae* Turcz.) berries: Bioactive components and antioxidant capacity. J. Food Sci. Technol..

[47] Alrahmany R., Avis T.J., Tsopmo A. (2013). Treatment of oat bran with carbohydrases increases soluble phenolic acid content and influences antioxidant and antimicrobial activities. Food Res. Int..

[48] Kitrytė V., Kraujalienė V., Šulniūtė V., Pukalskas A., Venskutonis P.R. (2017). Chokeberry pomace valorization into food ingredients by enzyme-assisted extraction: Process optimization and product characterization. Food Bioprod. Process..

[49] Radenkovs V., Juhnevica-Radenkova K., Kviesis J., Lazdina D., Valdovska A., Vallejo F., Lacis G. (2021). Lignocellulose-Degrading Enzymes: A Biotechnology Platform for Ferulic Acid Production from Agro-Industrial Side Streams. Foods.

[50] Tang Y., Zhang B., Li X., Chen P.X., Zhang H., Liu R., Tsao R. (2016). Bound Phenolics of Quinoa Seeds Released by Acid, Alkaline, and Enzymatic Treatments and Their Antioxidant and α-Glucosidase and Pancreatic Lipase Inhibitory Effects. J. Agric. Food Chem..

[51] Nguyen C.L., Nguyen H.V.H. (2018). The Quality of Mulberry Juice as Affected by Enzyme Treatments. Beverages.

[52] Sun T., Tang J., Powers J.R. (2005). Effect of Pectolytic Enzyme Preparations on the Phenolic Composition and Antioxidant Activity of Asparagus Juice. J. Agric. Food Chem..

[53] Shahidi F., Zhong Y. (2025). Lipid oxidation and improving the oxidative stability of food systems: A comprehensive review. J. Food Compos. Anal..

[54] Abeyrathne E.D.N.S., Nam K.C., Ahn D.U. (2021). Analytical methods for lipid oxidation and antioxidant capacity in food systems: A review. Antioxidants.

[55] Laguerre M., Bayrasy C., Panya A., Weiss J., McClements D.J., Lecomte J., Decker E.A., Villeneuve P. (2015). What makes good antioxidants in lipid-based systems? The next theories beyond the polar paradox. Crit. Rev. Food Sci. Nutr..

[56] Mozzon M., Foligni R., Mannozzi C., Vittori S. (2024). Assessment of lipid oxidation in fish and fish products processed by cold plasma technologies. Appl. Food Res..

[57] Suárez-Medina M.D., Sáez-Casado M.I., Martínez-Moya T., Rincón-Cervera M.Á. (2024). The Effect of Low Temperature Storage on the Lipid Quality of Fish, Either Alone or Combined with Alternative Preservation Technologies. Foods.

[58] de Camargo A.C., Regitano-D’Arce M.A.B., Rasera G.B., Canniatti-Brazaca S.G., do Prado-Silva L., Alvarenga V.O., Sant’Ana A.S., Shahidi F. (2017). Phenolic acids and flavonoids of peanut by-products: Antioxidant capacity and antimicrobial effects. Food Chem..

[59] Pinsirodom P., Namngam C., Taprap R., Nalinanon S., Thompson K.G., Puechkamutr Y. (2022). Mango Seed Kernel Extract as a Natural Antioxidant in Minced Fish During Frozen Storage. Curr. Appl. Sci. Technol..

[60] Dragoev S., Balev D., Ivanov G., Nikolova-Damyanova B., Grozdeva T., Filizov E., Vassilev K. (2014). Effect of superficial treatment with new natural antioxidant on salmon (*Salmo salar*) lipid oxidation. Acta Aliment..

