# Supplementary material for: Enzyme-Assisted Phenolic Extract of Murtilla Pomace: A Green Food Additive to Prevent Ozone-Induced Oxidation in Salmon
Source: Antioxidants (Basel). 2026 May 7;15(5):593. doi: 10.3390/antiox15050593 (PMC13203638; doi:10.3390/antiox15050593)

Table S1 - UPLC-ESI-MS/MS representative chromatograms of phenolic compounds in murtilla juice residue.

Compound	Representative chromatogram
3,4-Dihydroxybenzoic acid	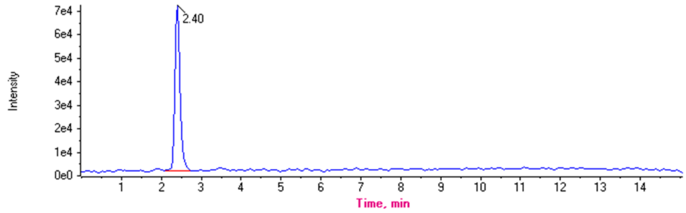
Gallic acid	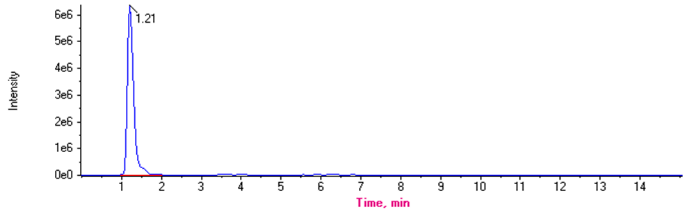
p-coumaric acid	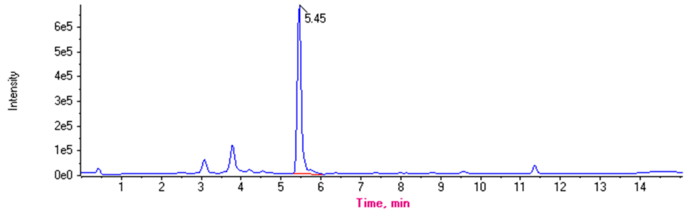
Vanillic acid	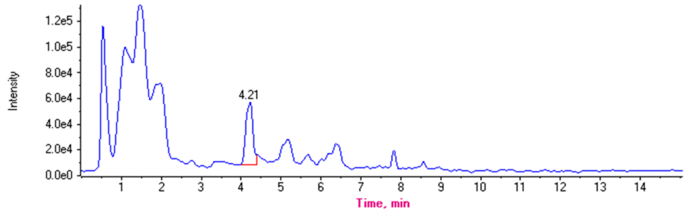
Caffeic acid	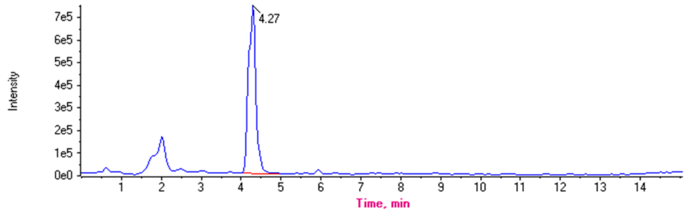
Ferulic acid	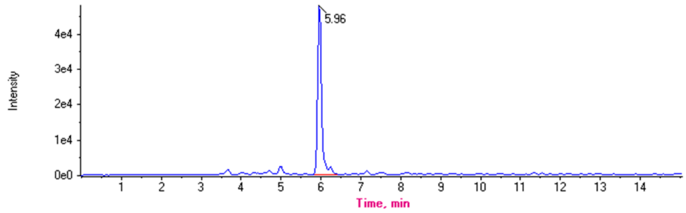
Syringic acid	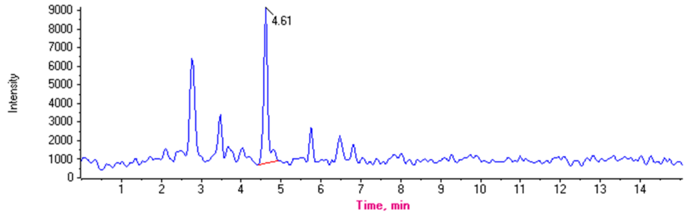
Catechin	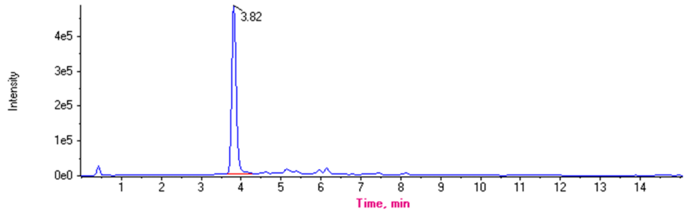

Epicatechin

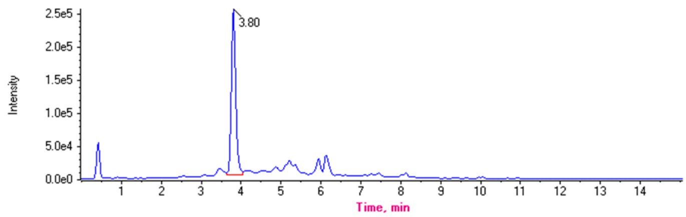

Quercetin

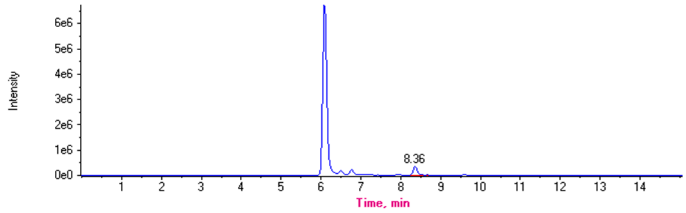

Miricetin

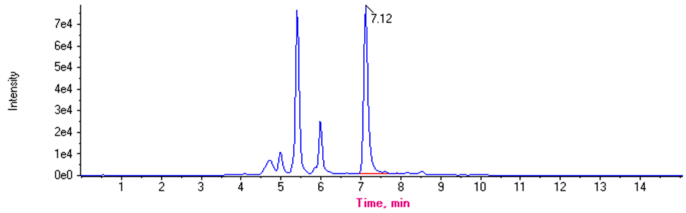

Quercitrin

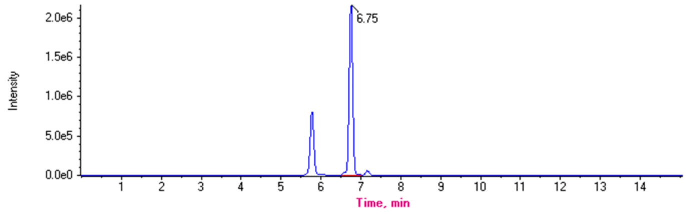

Taxifolin

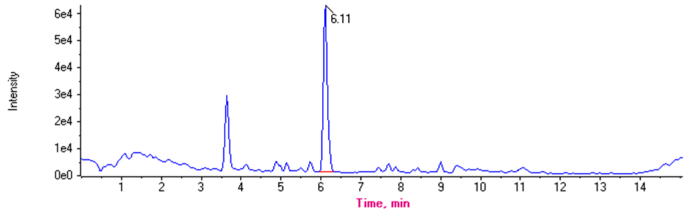

Rutin

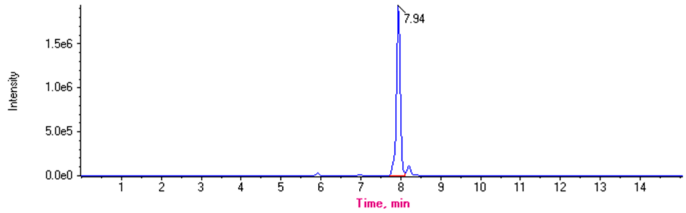

Supplement: Supplementary file 1 [file antioxidants-15-00593-s001.zip › antioxidants-4210821-supplementary.pdf]
